# Ceramide in cerebrovascular diseases

**DOI:** 10.3389/fncel.2023.1191609

**Published:** 2023-06-02

**Authors:** Huiqi Yuan, Bin Zhu, Cao Li, Zhigang Zhao

**Affiliations:** Department of Pharmacy, Beijing Tiantan Hospital, Capital Medical University, Beijing, China

**Keywords:** ceramide, cerebrovascular diseases, stroke, CSVD, endothelial cell, microglia, neuron

## Abstract

Ceramide, a bioactive sphingolipid, serves as an important second messenger in cell signal transduction. Under stressful conditions, it can be generated from *de novo* synthesis, sphingomyelin hydrolysis, and/or the salvage pathway. The brain is rich in lipids, and abnormal lipid levels are associated with a variety of brain disorders. Cerebrovascular diseases, which are mainly caused by abnormal cerebral blood flow and secondary neurological injury, are the leading causes of death and disability worldwide. There is a growing body of evidence for a close connection between elevated ceramide levels and cerebrovascular diseases, especially stroke and cerebral small vessel disease (CSVD). The increased ceramide has broad effects on different types of brain cells, including endothelial cells, microglia, and neurons. Therefore, strategies that reduce ceramide synthesis, such as modifying sphingomyelinase activity or the rate-limiting enzyme of the *de novo* synthesis pathway, serine palmitoyltransferase, may represent novel and promising therapeutic approaches to prevent or treat cerebrovascular injury-related diseases.

## 1. Introduction

Eukaryocyte use ∼5% of its gene and input substantial resource in synthesizing thousands of diverse structural and functional lipids (Sud et al., 2007), which are crucial for many physiological processes, such as energy storage in metabolism, providing fundamental structural elements of cell membranes, and acting as first or second messengers in signal transduction and molecular recognition processes (Mutlu et al., 2021). However, abnormal lipid storage plays a key role in many diseases. Among all lipid research, cholesterol and triacylglycerol are perhaps the most well-known, as they are widely used clinical biomarkers of metabolic disease and therapeutic intervention targets. In addition to cholesterol and triacylglycerol, recent studies have shown the pathological role of other lipid classes in multiple diseases, such as sphingolipids (Borodzicz-Jażdżyk et al., 2022; Li et al., 2022a; Mallela et al., 2022), glycolipid (Chen X. et al., 2022). Ceramide is a class of bioactive sphingolipid that consists of a fatty acid chain and a sphingosine backbone (Yu and Wang, 2022), with chain length ranging from 14 to 26 carbons, and it is the core molecule of sphingolipid metabolism. In addition to being fundamental constituents of cytomembranes, ceramides serve as key signaling mediators that regulate many cell processes, such as proliferation, survival, apoptosis, autophagy, and inflammation in health and disease, making it also known as a second messenger. Over the past decade, many studies have revealed a strong association between ceramide and the development of various diseases, including neurological disorders (Chowdhury et al., 2022; Custodia et al., 2022; Tringali and Giussani, 2022), cancer (Chung et al., 2021; Ghandour et al., 2021; Gomez-Larrauri et al., 2021; Sharma and Czarnota, 2022), cardiovascular disease (Shu et al., 2022; Zietzer et al., 2022), liver disease (Yu and Wang, 2022), kidney disease (Ueda, 2022), enteropatia (Li et al., 2022c), obesity (Kucuk et al., 2021), suggesting that it may be considered as a potential treatment target.

Cerebrovascular diseases, mainly caused by abnormal cerebral blood flow and secondary neurological injury, represent the leading causes of death and disability worldwide (Chen M. et al., 2022). The aging of the global population has dramatically increased the prevalence of cerebrovascular diseases, posing a significant burden on societies and healthcare systems worldwide. Stroke, especially ischemic stroke, is one of the most common cerebrovascular diseases, and it is the second leading cause of death and third leading cause of disability in adults worldwide (Campbell and Khatri, 2020; Aramburu-Núñez et al., 2022). According to World Health Organization estimates, more than 1.1 million people die from ischemic stroke every year (Chen et al., 2017), indicating a severe threat to human health. Cerebral small vessel disease (CSVD) is now recognized as a major cause of stroke and the most frequent reason for vascular dementia (Lam et al., 2023). CSVD is prevalent in the general population, affecting 5% of people aged 50 to almost 100% of people over 90 years old (Cannistraro et al., 2019; De Silva and Faraci, 2020). In addition, cerebrovascular diseases also include conditions such as carotid stenosis, cerebral aneurysms, and arteriovenous malformations, among others. Despite significant advances in cerebrovascular disease research in recent years, they remain difficult conditions to resolve due to a lack of knowledge on the pathophysiological mechanism. Therefore, a better understanding of the pathological mechanisms of cerebrovascular disease and the search for novel and effective targeting points have become the main research topics in the field.

Brain is known to contain the highest lipid level only next to adipose tissue, and disturbances in lipid metabolism have been associated with many age-related brain disorders. Ceramides accumulate in several tissues (e.g., liver and brain) during aging (Lightle et al., 2000; Cutler and Mattson, 2001; Trayssac et al., 2018), and emerging evidence has revealed that ceramide (Cer) is the most dysregulated lipid in cerebrovascular diseases, including stroke and CSVD (Gui et al., 2020; Ighodaro et al., 2020; Hannawi et al., 2021; Lee et al., 2022), and alterations in the regulation of ceramide pathobiology have been shown to impact the development of stroke in animal and cell models. Therefore, ceramide has been proposed as a promising biomarker for the early diagnosis and therapy of cerebrovascular diseases. Understanding the complex role of ceramide in cerebrovascular diseases requires adequate knowledge of ceramide biosynthesis. Therefore, the first section of this review provides an overview of the pathways involved in ceramide synthesis. After a brief description of ceramide, this review highlights their dysregulation in relation to stroke, CSVD, and related risk factors, focusing on the underlying mechanism in different types of stroke and CSVD.

## 2. Ceramide biosynthesis

**FIGURE 1:**
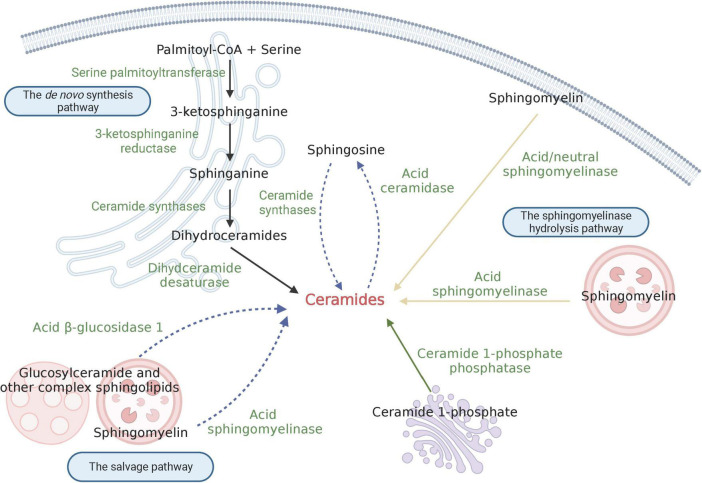
Schematic depiction of ceramide biosynthesis. The black solid arrow indicates the *de novo* synthesis pathway; the yellow solid arrow shows the sphingomyelinase hydrolysis pathway; and the blue dashed arrow signifies the salvage pathway. Created with BioRender.com.

Ceramides belong to a family of lipid molecules consisting of a sphingosine and a fatty acid linked by an amide bond (Fujii, 2021). There are many different kinds of ceramides that are differentiated by the combination of different acyl chain lengths of fatty acids with sphingosine, with non-hydroxy palmitic (C16:0) and stearic (C18:0) fatty acids being the most abundant (Pant et al., 2020). Ceramide is thought to be the core hub of sphingolipid metabolism and can affect key metabolic functions, and an imbalance of ceramide levels can contribute to various diseases. Currently, more than 30 enzymes implicated in ceramide metabolism have been reported, with highly conserved and strictly regulated processes (Gomez-Larrauri et al., 2021). Ceramide can mainly be differentiated into three major pathways: the *de novo* synthesis pathway, the sphingomyelinase hydrolysis pathway, and the salvage pathway (Figure 1). Additionally, ceramide can be synthesized through the dephosphorylation of ceramide 1-phosphate (C1P) by ceramide 1-phosphate phosphatase (CPP) (Custodia et al., 2021). C1P has been implicated in various functions, including proliferation/survival, migration, metabolism, and inflammation (Ouro et al., 2014; Rivera et al., 2015; Custodia et al., 2022).

The *de novo* synthesis pathway primarily occurs at the cytosolic leaflet of endoplasmic reticulum (ER); additionally, the presence of this pathway has also been found in mitochondria. Serine palmitoyltransferase (SPT) is the first enzyme involved in the *de novo* pathway, which synthesizes 3-ketosphinganine by condensating palmitoyl-CoA and serine. As the first-step enzyme, SPT is the major rate-limiting enzyme for the synthesis of ceramide. Subsequently, 3-ketosphinganine is reduced to produce sphinganine, which is catalyzed by 3-ketosphinganine reductase. Following this process, an acyl-CoA chain is bounded to sphinganine to form dihydroceramides, and this process is catalyzed by a group of ER-resident enzymes referred to as ceramide synthases (CerS) (Ho et al., 2022). To date, six isoforms of CerS (CerS1-6) have been identified in mammals (Kim et al., 2021). Each CerS has a specific fatty acyl substrate preference, resulting in the synthesis of dihydroceramides with specific acyl chain lengths. For instance, Ceramide Synthase 1 (CerS1) exhibits high specificity for C18-CoA substrates, thereby generating C18 dihydroceramide, while Ceramide Synthase 2 (CerS2) prefers to produce C22/24 dihydroceramide. More details on the substrate preferences of CerSs can be found in reviews by others (Ho et al., 2022; Shu et al., 2022). Besides, the distribution of CerS isoforms varies in different tissues. Apart from Ceramide Synthase 5 (CerS5) and Ceramide Synthase 6 (CerS6), which are expressed at relatively low levels in the vast majority of tissues, other CerSs are abundant in one or several types of tissues. CerS1 is highly expressed in mouse brain and skeletal muscles, but is almost undetectable in other kinds of tissues (Custodia et al., 2021); CerS2 is widely expressed in most mouse tissues; Ceramide Synthase 3 (CerS3) has the highest expression in mouse testis and skin; Ceramide Synthase 4 (CerS4) is highly expressed in mouse skin, heart, and liver (Ho et al., 2022). Finally, dihydroceramide desaturase converts dihydroceramide to form ceramide by introducing a double bond in position 4–5 trans. Once synthesized, ceramides are immediately transported to the Golgi apparatus by vesicular and non-vesicular mechanisms, where they can be further synthesized into complex sphingolipids, such as sphingomyelin and glucosylceramide.

The sphingomyelinase hydrolysis pathway occurs at the biomembrane, such as lysosomal membrane and plasma membrane. In this pathway, ceramide is derived from the hydrolysis of sphingomyelin (SM), which is catalyzed by sphingomyelinase. Under conditions of cellular stress, such as the presence of reactive oxygen species (ROS) (Dumitru et al., 2007), Tumor necrosis factor (TNF-α) (Edelmann et al., 2011), proinflammatory cytokine interleukin 1β (IL-1β) (Nalivaeva et al., 2000), viral and bacterial infections (Wangb et al., 2019), etc., sphingomyelinase becomes activated. This activation leads to the rapid hydrolysis of sphingomyelin into ceramide, which in turn mediates downstream signal transduction processes. Therefore, ceramide produced in the hydrolysis pathway is an important source of medicating cell-stress responses. According to the catalytic pH optimum, three subtypes of sphingomyelinase have been described, namely acid sphingomyelinase (ASM), neutral sphingomyelinase (NSM) and alkaline sphingomyelinase (alk-SM). ASM is one of the most studied sphingomyelinase and primarily localizes in lysosomes and the plasma membrane (Gorelik et al., 2016). It is generally believed that the optimal pH of ASM is between 4.5 to 5.0, but other study found that ASM can hydrolyze lipoprotein-sphingomyelin under conditions approximating neutral pH (Schissel et al., 1998). NSM has been detected in the nucleus, ER and plasma membrane (Clarke et al., 2006). Alk-SM is expressed in the intestinal villus and human bile (Cataldi et al., 2020). Unlike the other two sphingomyelinases, alk-SM can also hydrolyze platelet activating factor and lysophosphatidylcholine. Remarkably, ASM and NSM are implicated in signal transduction, whereas alk-SM plays a role in degradating sphingomyelin contained in the diet (Custodia et al., 2021).

The salvage pathway mainly occurs in acidic subcellular compartments like lysosomes or late endosomes, where the formation of ceramides is involved in the degradation of glucosylceramide, sphingomyelin, and other complex sphingolipids (Kitatani et al., 2008). Unlike the hydrolysis of sphingomyelin by ASM, glucosylceramide is converted to ceramide by the enzyme acid β-glucosidase 1 (Kitatani et al., 2009). Furthermore, ceramides produced by these processes can be broken down via the deacetylation of acid ceramidase to generating sphingosine base and a fatty acid chain (Kitatani et al., 2008). The sphingosine base can be released from the lysosome to the ER to provide the substrate for the *de novo* synthesis pathway. Thus, this pathway of sphingosine recirculation is termed the “salvage pathway.” Notably, the salvage pathway requires the action of a number of enzymes in acidic compartments. Defects in these enzymes, as seen in lysosomal diseases, may lead to dysfunction in the salvage pathway (Kitatani et al., 2008).

## 3. Ceramide in cerebrovascular diseases

The brain is known to be rich in sphingolipids, making it sensitive to changes in sphingolipid content. Ceramide, as the key precursor of all sphingolipids, serves as the central component of intracellular stress responses. There is a growing body of evidence from preclinical and clinical studies supporting a strong association between ceramide and cerebrovascular diseases (Table 1).

**TABLE 1:** Ceramide signaling in stroke and CSVD.

Species	Animal model/Disease	Changes in ceramide	Main observations after ceramide interference	References
Rats	24-h MCAO	Ceramide content increased by up to 595% in the ischemic core and 460% in perifocal or penumbral areas.	–	Takahashi et al., 2004
Mice	Photothrombo-sis	Ceramide levels in the peri-infarct cortex increased, and the elevated ceramides were derived from the *de novo* pathway.	The ASM inhibitor Fluoxetine did not alter ceramide concentration and improve functional outcome.	Brunkhorst et al., 2015
Rats	10-min 4-vessel occlusion	Accumulation of ceramide was observed in astrocytes and microglia in the CA1 and DG region.	The ASM inhibitor TPCK blocked the ceramide pathway.	Tian et al., 2009
Mice	30 min MCAO	Cerebral IR triggered accumulation of ceramide in brain during reperfusion phase, and the ceramide elevation was resulted from ceramide biosynthesis.	–	Yu et al., 2007
Mice	60 min MCAO	Cer (d18:1/18:0) accumulated over time in ischemic cores and surrounding areas.	–	Takatsugu et al., 2018
Rats	MCAO and bilateral common carotid arteries (CCAs)	Approximately a twofold increase in Cer (d18:1/16:0) and Cer (d18:2/16:0) in the peri-infarct region on post-stroke day 4.	–	Teppo et al., 2020
Mice	60 or 180 min MCAO	An increase of ceramide species, including C16:0, C20:0, C24:0 ceramides, were observed in the ischemic cerebral cortex.	Treatment with propranolol reduced brain ceramide accumulation, and improved functional outcome.	Luger et al., 2018
Mice	60 min MCAO	At 3 h post MCAO, ceramides exhibited an increase in levels of long-chain Cers but a decrease in very-long-chain Cers; at 24 h post MCAO, both long-chain and very long-chain Cers showed an increased trend.	–	Chao et al., 2019
Rats	120 min MCAO	Cer (d18:1/16:0), Cer (d18:1/18:0), and Cer (d18:0/18:0) were increased, while Cer (d18:1/24:1) was significantly downregulated.	Treatment with SPT inhibitor myriocin could reverse ceramide disturbances and decrease cerebral infarction, neurological deficits, and pathological changes in the cerebral cortex of rats.	Wang et al., 2022
Mice	4 h MCAO	–	Treatment with small molecule NSM inhibitor SMA-7 could reduce the size of cerebral infarcts.	Soeda et al., 2004
Rats	The endovascular perforation of the terminal ICA	A twofold increase in ceramide levels in the brain homogenates.	–	Testai et al., 2015
Humans	Acute ischemic stroke	C14:0, C24:0 and C20:0 Cer were increased, while C24:1 Cer were diminished in patients after stroke.	–	Fiedorowicz et al., 2019
Humans	Acute ischemic stroke	Plasma levels of C16:0, C22:0, and C24:0 ceramides in stroke patients were higher than in those control cases, and higher levels of ceramides were associated with higher risk of the clinical severity of ischemic stroke.	–	Gui et al., 2020
Humans	Acute ischemic stroke	AIS patients had significantly higher plasma levels of ceramides (C14-20), ceramides (>C22), were significantly lower in AIS patients. Higher levels of Cer were significantly associated with poor functional outcomes.	–	Lee et al., 2022
Humans	Acute ischemic stroke	Cer (d18:1/16:0), Cer (d18:1/18:0), Cer (d18:1/20:0), Cer (d18:1/24:1) were significantly higher in AIS patients.	–	Lee et al., 2023
Humans	SAH	SAH patients had an approximately four fold increase in total Cer and DHC levels.	–	Testai et al., 2015
Humans	SAH	Compared to control subjects, SAH patients exhibited higher total ceramide levels in their cerebrospinal fluid. Moreover, patients with symptomatic vasospasm had increased ceramide levels, particularly C18:0 Cer, compared to those with asymptomatic vasospasm.	–	Testai et al., 2012
Humans	CSVD	Cer (d34:2), Cer (d36:4), Cer (d16:0/18:1), Cer (d38:6), Cer (d36:3) and Cer (d32:0) were increased in CSVD patients.	–	You et al., 2020
Humans	CSVD	Subcortical microstructural white matter disruption was associated with elevated serum ceramides (C18 and C20) and reduced manual dexterity in a population with CSVD.	–	Hannawi et al., 2021
Humans	CSVD	Cer 34:1 correlated with WMH load.	–	Azizkhanian et al., 2019
Humans	CSVD	Increased in the ceramide C16:0/24:0 ratio significantly related to greater odds of any CMB or of a lobar CMB.	–	Ighodaro et al., 2020

### 3.1. Stroke

Stroke is defined as a neurological deficit resulting from acute focal damage to the central nervous system (CNS), initiated by a vascular cause (Campbell and Khatri, 2020). Stroke can be either ischemic or hemorrhagic. Ischemic stroke caused by arterial occlusion is responsible for the majority of strokes (Campbell et al., 2019). Current treatments for ischemic stroke attempt to recover reperfusion by intravenous thrombolysis and endovascular thrombectomy. These approaches do improve disability rates, but time is critical, which creates a stressful burden on the health-care system. Hemorrhagic stroke accounts for a small proportion of strokes but is associated with severe morbidity and high mortality due to sudden deterioration of consciousness and neurological dysfunction (Smith and Eskey, 2011). Vasospasm is a frequently encountered complication of subarachnoid hemorrhage (SAH) and a major determinant of outcome (Crowley et al., 2011). Although the use of drugs can effectively reverse vasospasm, they do not improve SAH-associated mortality. As a result, there is an urgent need for new non-invasive approaches or drug treatments to prevent or delay stroke progression in order to improve the quality of life for patients and reduce mortality.

Several stroke animal models have demonstrated the increased presence of ceramide species in the ischemic/reperfusion (IR) and hemorrhagic injured brain. For example, in spontaneously hypertensive rats subjected to 24-h middle cerebral artery occlusion (MCAO), brain ceramide content increased by up to 595% in the ischemic core and 460% in perifocal/penumbral areas, respectively (Takahashi et al., 2004). In a photothrombosis stroke model, the total ceramide levels in the peri-infarct cortex decreased at day 1 but significantly increased from day 3 to day 7, eventually returning to normal levels at day 28 (Brunkhorst et al., 2015). In rats subjected to transient cerebral ischemia by 4-vessel occlusion, accumulation of ceramide was observed in astrocytes and microglia in the Cortical Area 1 (CA1) and Dentate Gyrus (DG) region after ischemic stimuli (Tian et al., 2009). Interestingly, the accumulation of ceramide species seems to occur during the reperfusion phase, as there were no changes in ceramide levels after ischemia without reperfusion (Yu et al., 2007). In the endovascular perforation of the terminal internal carotid artery (ICA) rat model, the ceramide level of brain homogenates was found to be twofold higher compared to the control group (Testai et al., 2015). Furthermore, the increased ceramide species were mainly endogenous long chain ceramides, such as C16, C18, C20 ceramide. Interestingly, aging, a significant contributing factor to stroke, also appears to be associated with the accumulation of long-chain ceramides. A study discovered that plasma C16:0 ceramide is linked to slower gait, an important aging marker of physical function (Wennberg et al., 2018). Furthermore, the accumulation of long-chain C16 and C18 ceramides was observed in the mitochondria of aged rats (Monette et al., 2011). In mice suffering from transient middle cerebral artery occlusion (tMCAO) and reperfusion for 60 min, Cer (d18:1/18:0) accumulated over time in ischemic cores and surrounding areas following ischemia onset (Takatsugu et al., 2018). In the peri-infarct region on post-stroke day 4, metabolomics data showed approximately a 2-fold increase in Cer (d18:1/16:0) and Cer (d18:2/16:0) (Teppo et al., 2020). Distinct trends were observed between long-chain and very-long-chain ceramides (with acyl chain length ≥ 22) in cerebral ischemic injury. Very-long-chain ceramides, including Cer (18:1/23:0) and Cer (18:1/24:0), were significantly decreased at 3 h post-MCAO (Chao et al., 2019). This finding was also confirmed by another experiment using ultra-performance liquid chromatograph quadrupole time-of-flight mass spectrometry (UPLC-Q-TOF/MS)–based lipidomic analysis to identify disordered lipid metabolites in stroke, where Cer (d18:1/16:0), Cer (d18:1/18:0), and Cer (d18:0/18:0) were significantly increased, while very-long-chain Cer (d18:1/24:1) was significantly downregulated after MCAO (Wang et al., 2022).

The pathway of the source of ceramide in stroke remains inconclusive, as evidence has been found for both the *de novo* synthesis and the sphingomyelinase hydrolysis pathways. Several studies suggest that the increased ceramide level is derived from the *de novo* pathway, as dihydroceramide, the direct ceramide precursor of the *de novo* pathway, was correspondingly increased with ceramide (Yu et al., 2007; Brunkhorst et al., 2015), but changes in ASM and NSM activity or the content of sphingomyelin species could not be detected (Yu et al., 2007). Treatment with myriocin, a serine palmitoyltransferase (SPT) inhibitor that affects the *de novo* synthesis pathway, can reverse ceramide disturbances, attenuating cerebral I/R injury (Wang et al., 2022). Additionally, ceramide synthase activity is altered during stroke (Ouro et al., 2022), which seems to provide further evidence supporting the idea that ceramides originate from the *de novo* synthesis pathway. Mitochondrial ceramide synthase activity increased after IR stimulation (Yu et al., 2007). Another study that supports this point investigated the role of Sirtuins 3 (SIRT3) in regulating the mitochondrial ceramide synthase response to cerebral IR injury (Novgorodov et al., 2016). SIRT3 is a primary mitochondrial deacetylase involved in the regulation of metabolism and mitochondrial homeostasis. Cerebral IR injury triggers ceramide accumulation, mitochondrial dysfunction, and the generation of ROS, ultimately promoting brain damage. SIRT3 knockout could reverse ceramide accumulation and brain damage by increasing the acetylation of CerS1, 2 and 6, suggesting that the increased ceramide is derived from the *de novo* synthesis pathway. Yet some researchers believe that ceramide production in ischemia injury occurs through the sphingomyelinase pathway, but not through the *de novo* pathway (Tian et al., 2009). In one study, serum/glucose deprivation induced rapid activation of NSM, leading to an increase in ceramide content. Treatment with small molecule NSM inhibitors could inhibit the increase in ceramide levels and significantly reduce the size of cerebral infarcts (Soeda et al., 2004).

Contrary to endogenous long chain ceramides, exogenous short chain ceramides seem to play a beneficial role in stroke. These short chain ceramides such as C2 and C8 ceramides are a class of synthetic, cell-permeable ceramides. Sublethal stress stimuli such as transient global or focal ischemic or systemic inflammation treatment can induce tolerance to experimental hypoxia and stroke, leading to decreased infarct size (Takahashi et al., 2004). TNF-α is essential to that process as treatment with TNF-α-binding protein can abolish tolerance. Ceramide is a downstream messenger in the TNF-α signaling pathway. It has been discovered that preconditioning C2-ceramide by intraventricular injection can reduce hypoxic-ischemic-induced brain infarct volume by 45–65% in neonatal rat (Chen et al., 2001). A similar view was confirmed in another study in which pretreatment with C2- and C8-ceramide infusion could significantly decrease infarct volumes by approximately 14 and 17%, respectively (Furuya et al., 2001). Additionally, phytoceramide also has a beneficial effect on focal transient ischemic brain damage. Orally administered phytoceramide significantly reduced MCAO/reperfusion-induced brain infarction and edema, as well as the development of behavioral disabilities in animals (Lee et al., 2015).

A series of research studies have revealed the relationship between ceramide and stroke patients. In acute ischemic stroke patients, long-chain ceramides were significantly increased, and very-long-chain ceramides were significantly decreased compared to non-stroke controls (Fiedorowicz et al., 2019). Higher levels of ceramides were associated with a higher risk of the clinical severity of ischemic stroke (Gui et al., 2020). Importantly, higher levels of Cer (d18:1/18:0), Cer (d18:1/20:0), and Cer (d18:1/22:0) were significantly associated with poor functional outcomes (Lee et al., 2022). In another study, very-long-chain ceramide Cer (d18:1/24:1) was significantly higher, like other long-chain ceramides, than those in the controls (Lee et al., 2023). In addition, there is a notable association between ceramide and hemorrhagic stroke (Altura et al., 2020). Compared to control subjects, subarachnoid hemorrhage (SAH) patients exhibited higher total ceramide levels in their cerebrospinal fluid. Moreover, patients with symptomatic vasospasm had increased ceramide levels, particularly C18:0 Cer, compared to those with asymptomatic vasospasm (Testai et al., 2012). Furthermore, ceramide is correlated with the prognosis following stroke. The ceramide- and phospholipid-based cardiovascular risk score (CERT2) has been found to predict the risk of cardiovascular disease events, which consists of four ceramide/ceramide or ceramide/phosphatidylcholine ratios. A study showed that the risk score CERT2 is strongly correlated with stroke mortality in the elderly (Katajamäki et al., 2022). Depression is a neuropsychiatric disorder often associated with stroke. It has been reported that approximately 85% of stroke patients have the possibility of developing depression (Hackett and Pickles, 2014). A recent study found that post-stroke depression (PSD) patients had significantly higher levels of serum ceramides, including C16:0, C18:0, C24:0, C24:1 Cer compared to non-post-stroke depression (Non-PSD) patients (Hong et al., 2021).

### 3.2. CSVD

Cerebral small vessel disease is a class of pathological processes characterized by damages to small arteries, arterioles, capillaries, and small veins of the brain (Markus and Erik de Leeuw, 2023). CSVD is the leading vascular cause of cognitive impairment and dementia, and can also results in motor and balance impairment, as well as behavioral symptoms like depression and apathy (Markus and Erik de Leeuw, 2023). Consequently, CSVD is a dominant risk factor for transition to disability, and recent data demonstrate that it also pose a significant health burden in low- and middle-income countries (Lam et al., 2023). Despite the significance and concern of CSVD, current preventative or therapeutic approaches are quite limited. So far, the most widely accepted treatment is to control vascular risk factors that can be intervened with and change unhealthy lifestyles (Gao et al., 2022). Consequently, there is a pressing need to discover novel approaches to delay the clinical manifestations caused by CSVD.

In a study involving 20 patients with age-related CSVD and 14 controls, ceramides including Cer (d34:2), Cer (d36:4), Cer (d16:0/18:1), Cer (d38:6), Cer (d36:3), and Cer (d32:0) were found to be increased compared to controls (You et al., 2020). White matter hyperintensities (WMHs) seen on brain magnetic resonance imaging (MRI) are the most common imaging characteristics of CSVD (Wardlaw et al., 2013). Higher WMHs load is always associated with worse cognition impairment, especially executive function, processing speed, and episodic memory (Yang et al., 2022). Ceramides are highly enriched in the human white matter tracts, and elevated ceramide levels are believed to be involved in subcortical white matter injury in the cognitively normal elderly (Gonzalez et al., 2016). In subjects with CSVD, elevated serum ceramides, especially C18 and C20 ceramides, are also associated with subcortical microstructural white matter disruption (Hannawi et al., 2021). Another cohort of 58 patients who underwent an MRI but did not have a clinical stroke event were analyzed for plasma sphingolipid levels (Azizkhanian et al., 2019). According to the research findings, two brain-specific sphingolipids, SM 38:1 and Cer 34:1, were significantly correlated with high Fazekas scoring (FS), a method used to evaluate the degree of WMH. Additionally, lesions of CSVD are also related to changes in ceramide. Cerebral microbleeds (CMBs) and lacunes are common lesions of CSVD. In a cohort of 548 individuals, the association between plasma ceramide levels and the presence and number of CMBs and the presence of lacunes were elucidated (Ighodaro et al., 2020). Logistic regression models adjusted for age, sex, hypertension, and diabetes mellitus showed that an increase in the ceramide C16:0/24:0 ratio was significantly related to greater odds of any CMB or a lobar CMB, and that association was stronger for women, perhaps due to most ceramide levels being higher among women than men. Vascular dementia (VaD) refers to complex neurocognitive impairment secondary to multiple cerebrovascular lesions (Liu et al., 2020) including CSVD. In contrast to several other studies, a study that recruited 48 VaD patients and 49 age- and sex-matched cognitively normal controls found that both long-chain ceramide and very-long-chain ceramide were lower in VaD patients (Liu et al., 2020).

### 3.3. Cerebrovascular diseases risk factors

Cerebrovascular diseases, including stroke and CSVD, share many risk factors with other cardiovascular diseases, although their relative importance may vary. The most potent risk factor for vascular injury is hypertension. In addition, diabetes, hyperlipidemia, and physical inactivity are also significant risk factors. Many studies have provided evidence linking ceramide to the risk factors of cerebrovascular diseases.

Hypertension is a leading risk factor for vascular disease and mortality worldwide. In patients with essential hypertension, plasma ceramide levels were significantly increased compared to healthy normotensive controls, and ceramide levels were correlated with the severity of hypertension. In the Uppsala seniors cohort study, which involved 504 individuals with blood pressure (BP) measurements and metabolomics profiling during a 5-years follow-up examination, the level of ceramide (d18:1,C24:0) had a positive relation with continuous diastolic blood pressure (DBP) change (Lin et al., 2020). Furthermore, the relationship between ceramide and hypertension was also observed in spontaneously hypertensive rats (SHR), where C16:0, C22:0, C24:1, and C24:0 ceramide mainly contributed to the elevated ceramide level (Spijkers et al., 2011b). Moreover, antihypertensive therapy might be linked to lowering the ceramide level. Losartan treatment could reduce arterial contractions by lowering vascular ceramide levels in SHR (Spijkers et al., 2011a). *Uncaria*, a traditional Chinese medicine used for treating high blood pressure in China for many decades, was found to markedly affected ceramide levels in SHR, indicating that ceramide might be a key effector in the hypotensive action of *Uncaria* (Liu et al., 2018).

Diabetes mellitus (DM) is another risk factor for vascular injury. Evidence is emerging that ceramide serves as a particularly important contributor to insulin resistance and DM complications. In a study involving male and female obese populations, each gender was divided into three groups: Normal Glucose Tolerance (NGT), Impaired Glucose Tolerance (IGT), and Type 2 Diabetes subjects (T2D). The findings revealed that total hepatic ceramides, especially C16:0-Cer, C18:0-Cer, C22:0-Cer, and C24:0-Cer, were significantly increased in T2D compared to NGT in both male and female obese individuals. However, in the male obese population, total ceramides were also significantly increased in the IGT group compared to the NGT group (Razak Hady et al., 2019). This is consistent with another study that found C16 Cer levels were significantly increased in obese-diabetic patients compared to obese-non-diabetic patients, indicating that C16 Cer plays a pivotal role in inducing insulin resistance (Chathoth et al., 2022). In a prospective population-based cohort that investigated the cross-sectional and longitudinal relationship of baseline ceramides with prevalent and incident diabetes mellitus type 2 (T2DM), ceramides (C16:0, C18:0, C18:0/C16:0 ratio, C18:0/C24:0 ratio) were found to be associated with prevalent T2DM, and ceramides (C18:0, C18:0/C16:0 ratio) were related to incident T2DM (Dugani et al., 2021). In patients with comorbid acute coronary syndrome and type 2 diabetes mellitus (ACS-DM), their circulating ceramide levels, including Cer(d18:1/16:0), Cer(d18:1/18:0), and Cer(d18:1/24:1), were higher compared with acute coronary syndrome (ACS) patients. Pearson correlation coefficients analysis indicated there were relatively strong correlations of Cer(d18:1/18:0) and Cer(d18:1/24:1) with fasting blood glucose levels (Cao et al., 2020). In a case-control study involving 29 gestational diabetes mellitus (GDM) patients and 63 healthy controls, C24:0 ceramide concentration was significantly lower in the GDM compared to the control group (Lantzanaki et al., 2022). Additionally, the relationship of ceramide and DM is also implicated in animal disease models. In an obese T2DM rat model, blood ceramide levels were significantly increased, and treat with Gegen Qinlian Decoction could decrease the ceramide level and improve diabetes-related symptoms (Zhou et al., 2021). Furthermore, inhibiting ceramide levels through pharmacological or genetic approaches has been shown to improve insulin resistance in obese animal models. This indicates that ceramide accumulation contributes to impaired insulin sensitivity (Imierska et al., 2022; Li et al., 2022d; Xia et al., 2022).

Hyperlipidemia is associated with a 28% increased risk of ischemic stroke, which is behind hypertension, diabetes, and smoking as a critical risk factor for stroke, and it is a key therapeutic target for stroke risk modification (Brooks and Schindler, 2019). Hepatic steatosis (HS) is usually accompanied by dyslipidemia. In a study that assessed the association between plasma ceramides and HS in adolescents, significant correlations were observed between C14:0, C16:0, C18:0, C24:0 Cers and higher levels of cholesterol (Maldonado-Hernández et al., 2017). Bariatric surgery is associated with near immediate remission of hyperlipidemia. In 3 and 6 months post-surgery, total plasma ceramide concentrations were reduced which accompanied by a reduction in lipid levels (Huang et al., 2011). High-fat diet (HFD) feeding is a common approach to inducing hyperlipemia. In mice fed with HFD, ceramide concentrations in serum and liver were significantly increased, and that were consistent with the trend of serum triglyceride, total cholesterol, and low-density lipoprotein cholesterol levels (Si et al., 2020; Jiang et al., 2021; Jiang X. et al., 2022). In addition, ceramide levels were elevated in estrogen-deficiency-induced hyperlipidemia rats (Vinayavekhin et al., 2016) and experimental hyperlipemia beagles (Qu et al., 2022). *In vitro*, cells exposed to hyperlipidemia simulation (saturated fatty acid-albumin complexes) could induce ceramide production (Ntika et al., 2019).

In addition to the common risk factors mentioned above, liver disease may be an unrecognized or easily confused risk factor for stroke. Non-alcoholic fatty liver disease (NAFLD) is the most frequent chronic liver disease, affecting approximately 25% of the general population (Younossi et al., 2016). Although NAFLD is more prevalent in patients with major stroke risk factors (e.g., diabetes mellitus, obesity, and hypertension), the relationship between NAFLD and stroke appears to be independent of these risk factors (Alkagiet et al., 2018). Interestingly, several lines of evidence link NAFLD to changes in ceramide levels (Simon et al., 2019). Almost all ceramide species were significantly increased in non-alcoholic hepatosteatosis (NASH, the more advanced stage of NAFLD) (Luukkonen et al., 2016; Yang et al., 2019). Dietary weight loss is considered essential for NASH treatment. Weight loss in NASH patients through a 1-year course of lifestyle changes significantly reduced serum ceramide levels, suggesting that ceramide levels are important factors in the development of NAFLD (Promrat et al., 2011). Deregulation of ceramide metabolism in the liver during NAFLD, has also been documented in experimental animal models. In ob/ob mice, a type of transgenic animal model of NAFLD, also manifest with ceramide increases in correlation with the degree of steatosis (Yetukuri et al., 2007). In HFD-fed rats, Myriocin (an SPT inhibitor) reversed elevated body weight and serum transaminases, alleviated dyslipidemia, and significantly attenuated liver steatosis-related pathology (Yang et al., 2019). Moreover, ASM is activated in NASH, and ASM deficiency confers resistance to hepatic steatosis mediated by an HFD or methionine-choline deficient diet (Fucho et al., 2014).

## 4. Ceramide in different types cells in cerebrovascular diseases

**FIGURE 2:**
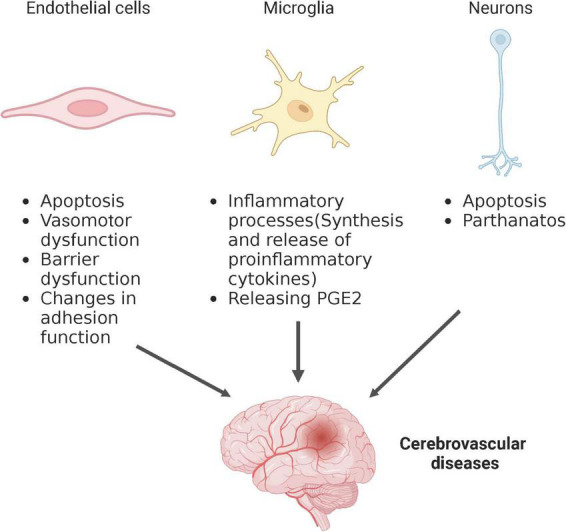
Schematic depiction of ceramide regulation of different cell types affecting cerebrovascular disease. Created with BioRender.com.

The brain is considered to have highly metabolic demand, responsible for approximate 20% of the body’s oxygen and glucose consumption (Gonçalves et al., 2023). Glucose and oxygen supply to the CNS are transported by blood flow, making a healthy vascular system critical for normal brain function. The brain contains diverse types of cells that work together to maintain normal cerebral physiological function, and related cell injury or dysfunction can lead to abnormal cerebral blood flow and secondary neurological injury, implicating them in the pathological process of cerebrovascular diseases. Ceramides may be potential therapeutic targets for cerebrovascular diseases, although the mechanisms by which ceramide affects different types of brain cells’ functions are not yet completely understood. Several mechanisms, described in other disease model, may also be involved in cerebrovascular diseases. Therefore, to better understand the pathogenesis of cerebrovascular disease, this section will summarize the underlying mechanisms of the relationship between ceramide and brain cell function, including but not limited to cerebrovascular diseases (Figure 2).

### 4.1. Endothelial cells

Endothelial cells are a single layer of flat epithelial cells located on the inner surface of the vascular lumen (Huang Y. et al., 2022). They are distributed throughout the large blood vessels and microvessels of the brain and maintain normal vascular function. Endothelial injury, including death and dysfunction, is seen in a variety of cerebrovascular diseases, including stroke and CSVD, and is considered the initial event in the development of these diseases. Endothelial cell death, mainly apoptosis, is the immediate cause of vascular dysfunction. It has been confirmed that ceramide can directly induce endothelial cell apoptosis *in vitro* and *in vivo* by forming the lipid signal transduction platform of apoptosis-related pathways (Matsunaga et al., 2004; Chen et al., 2005, 2013; Niaudet et al., 2017; Takehara et al., 2020). Studies in recent years have found that ceramide induces endothelial injury in a non-apoptotic manner as well.

The loss of vasomotor function is attributable to the decline in endothelial nitric oxide synthases (eNOS) activity and nitric oxide (NO) bioavailability. eNOS is essential for NO generation (Zhou et al., 2022), and its activity is principally govern by phosphorylation/dephosphorylation of the threonine 494 (T494) and serine 1176 (S1176) residues. Protein phosphatase 2A (PP2A) is a ceramide-activated phosphatase that directly decreases eNOS phosphorylation at Ser1177 residue, leading to deactivation of eNOS. Studies have shown ceramide regulates eNOS activity or NO bioavailability in two ways. On the one hand, ceramide can increase the bond of PP2A with eNOS and decrease the bond between eNOS and protein kinase B (Akt) and between eNOS and heat shock protein 90 (Hsp90), then PP2A promotes the dephosphorylation of Akt that colocalizes with eNOS and/or dephosphorylates eNOS at S1176 directly (Smith et al., 2006; Zhang et al., 2012). On the other hand, ceramide produces oxidative stress and increases the ROS level via nicotinamide adenine dinucleotide phosphate (NADPH) oxidase-dependent manner, which can rapidly destroy bioactive NO (Li et al., 2002). The underlying mechanism of the effect of ceramide on endothelial barrier dysfunction may depend on ceramides that derived from different pathways. In the early stages of injury, the increased ceramide level is derived from ASM activation, which results in an increase in calcium influx and Ras homolog gene family, member A (RhoA) activation, leading to canonical transient receptor potential channel 6 (TRPC6) overexpression, ultimately leading to cytoskeletal rearrangement and the breakdown of intercellular junctions. In the late stages of injury, ceramide from paracellular cells contributes to the *de novo* synthesis of ceramide in endothelial cells, inducing apoptosis and resulting in barrier dysfunction. The underlying mechanism is linked to the thioredoxin-interacting protein (TXNIP)/thioredoxin (TRX)/apoptosis signal regulating kinase-1 (ASK1)/p38 and c-Jun N-terminal kinase (JNK) pathways (Jiang J. et al., 2022). Besides, the disruption of endothelial cell barrier function induced by accumulated ceramide may be involved in the degradation of zonula occludens-1 (ZO-1) and zonula occludens-2 (ZO-2) (Becker et al., 2018), which are essential for the structural and regulatory functions of tight junctions. The association of ceramide and adhesion function has also been reported. Blood-derived immune cells can enter the brain and cause local inflammation, leading to the loss of vascular autoregulation. Upon ASM-derived ceramide accumulation, the phosphorylation of ezrin increases, as well as the interaction between filamin and intercellular adhesion molecule-1 (ICAM-1) upon ICAM-1 clustering, which leads to increasing microvilli formation and facilitating the transendothelial migration of T cells (Lopes Pinheiro et al., 2016). Unlike the above studies, two studies revealed the positive effect of ceramide on endothelial cell function. Annexin A5 is an essential element of membrane repair machinery. High glucose treatment, a risk factor for cerebrovascular diseases, decreases the association of membrane ceramide with annexin A5, impairing instant cell membrane reseal (ICMR) and promoting vascular inflammation (Chen Y. et al., 2022). Ceramide might also participate in the regulation of stroke recovery. A study showed that inhibition of ASM/ceramide could induce the formation and release of small extracellular vesicles (sEVs) to promote angiogenesis. Increased angiogenesis further promotes brain remodeling via increased blood-brain barrier integrity, reduced leukocyte infiltrates, and increased neuronal survival (Mohamud Yusuf et al., 2022).

### 4.2. Microglia

Microglia, as resident immunocompetent cells in the brain, are the main cells that respond to pathophysiological changes caused by cerebrovascular diseases. Stimulators in injury areas such as ROS, extracellular ATP, and NO mediate the chemotaxis and activation of microglia. Once activated, microglia releases inflammatory mediators, which can affect neurotransmission, mediate neurotoxicity and inducing apoptosis, then directly influence behavior and brain injury (Lu et al., 2021). Studies have shown that ceramide is involved in the inflammatory respond of microglia during cerebrovascular diseases.

The cytokine IL-1β is a primary mediator of inflammatory processes within the CNS and is released by activated microglia, which can exacerbate brain injury at elevated levels. Ceramide has been implicated not only in IL-1β generation but also in IL-1β secretion. The effect of ceramide on IL-1β generation might be related to regulating the NOD-like receptor thermal protein domain associated protein 3 (NLRP3) inflammasome, a protein complex consisting of NLRP3, apoptosis-associated speck-like protein (ASC), and caspase-1, which is involved in the inflammatory response by producing IL-1β. Palmitate (PA), a cerebrovascular disease risk factor, induces ceramide increase and subsequently releases IL-1β in microglia. Exposure of microglia to the *de novo* ceramide synthesis inhibitor significantly prevented PA induced ASC speck formation and IL-1β secretion, and this was abolished in ASC^–/–^ mice (Scheiblich et al., 2017), indicating that ceramide serves as the intracellular modulator of NLRP3 inflammasome assembly to mediate the IL-1β generation in microglia. Apart from directly exporting IL-1β, microglia can also release IL-1β through microvesicles (MVs). IL-1β contained in MVs can deliver the cytokine to long distance from the donor microglia, in possible proximity to IL-1β receptors present on target cells, thus avoiding dispersal and dilution of the cytokine in the extracellular environment. Inflammatory stimuli induce the translocation of ASM to the cytomembrane outer leaflet to generate ceramide, thereby inducing budding of IL-1β contained-MVs. The formation of blebs is likely caused by redistribution of extracellularly hydrolyzed-ceramide within the bilayer and by local enrichment of the cone-shaped sphingolipid into the inner leaflet of the membrane (Turola et al., 2012). Prostaglandin E2 (PGE2) is able to induce neuronal apoptosis and increase intracellular level of ROS, and plays an important role in the pathological process of cerebrovascular diseases. Microglia contribute significantly to the release of prostaglandins (PGs) during neuronal insults. Cyclooxygenase-2 (COX-2) is the major enzyme for the synthesis of PGE2. Ceramide can activate p38 mitogen-activated protein kinase (MAPK) directly, which is involved in providing mRNA stability and COX-2 expression, thereby releasing PGE2 and amplifying neuroinflammatory events (Akundi et al., 2005).

Besides its detrimental effects on nerve injury, ceramide in microglia may have beneficial effects after stroke. After cerebral ischemia, ceramide can induce microglial cell-cycle arrest by up-regulating cyclin-dependent kinase (Cdk) inhibitors p21 and p27 through activation of PP2A, thereby preventing glial scar formation and reducing the release of inflammatory factors (Adibhatla and Hatcher, 2010; Gusain et al., 2012). Administration of C2 ceramide suppressed microglial activation induced by lipopolysaccharide (LPS) through the inhibition of the ROS, MAPKs, phosphoinositide 3-kinase (PI3K)/Akt, and Janus kinase (Jak)/signal transducers and activators of transcription (STAT) pathways with upregulation of protein kinase A (PKA) and heme oxygenase-1 expressions. Toll like receptor 4 (TLR4), expressed on the surface of microglia, plays a crucial role in activating Nuclear factor-kappa B (NF-κB), which in turn contributes significantly to the pro-inflammatory response of microglia (Li et al., 2022b). Interestingly, C2 ceramide could interfere with LPS and TLR4 interactions, thereby inhibiting TLR4 signaling and subsequent inflammation (Jung et al., 2013). Brain-Derived Neurotrophic Factor (BDNF) is a member of the neurotrophin family, which is involved in neuroprotection, neurogenesis and neuroplasticity, and has been identified as a key regulator of motor learning and rehabilitation after stroke (Mang et al., 2013). A study showed that C8-ceramide promoted BDNF secretion in microglia via protein kinase Cδ (PKCδ) and/or ε signaling pathway (Nakajima et al., 2002) thereby accelerating post-stroke repairing.

### 4.3. Neurons

Neurons are highly sensitive to the lack of glucose and ATP, and they are the first brain cells to die in the area affected by the lack of blood flow during cerebrovascular diseases. In ischemic brain cells, neuron injury and death are the main cause of neurological deficit. Some research has shown that the effect of ceramide on cerebrovascular diseases might be associated with neuron death, especially apoptosis (Yu et al., 2000; Soeda et al., 2004; Wang et al., 2022). Interestingly, ceramide can both induce and protect neurons from apoptosis. NGF (nerve growth factor) deprivation is the main cause of neuron death. The progression of a cell through the G1 and S phases, which facilitating apoptosis, requires phosphorylation of retinoblastoma protein (pRb). Conversely, dephosphorylation of pRb favors cell cycle arrest, thereby inhibiting apoptosis. Short chain ceramides, such as C6-ceramide, can inhibit neurons apoptosis induced by NGF deprivation via activating protein phosphatases 1 (PP-1), and the activated PP-1 prevents the hyperphosphorylation of pRb (Plummer et al., 2005). Another short chain ceramides, C2-ceramide, has been reported to induce neuronal apoptosis. This effect is correlated with differential modulation of mitogen-activated protein kinase (MAPK) cascades. Among MAPK cascades, p38 is an essential kinases involved in ceramide-induced apoptosis by increasing p38 phosphorylation (Willaime et al., 2001). Besides apoptosis, ceramide might induce neuron death by a novel manner, namely parthanatos. Parthanatos, also known as Poly(ADP-ribose) (PAR) polymerase-1 (PARP-1)-dependent cell death, is characterized by hyperactivation of PARP-1, accumulation of PAR polymers, and apoptosis-inducing factor (AIF) nuclear translocation from mitochondria, thereby resulting in large DNA fragments and chromatin condensation, and ultimately, cell death (Huang P. et al., 2022). A recent study found that ceramide can induce PARP-1 overactivation, increased PAR polymer levels, facilitate AIF and migration inhibitory factor (MIF) nuclear translocation, and induce DNA damage and neuron death by increasing ROS levels (Fan et al., 2022).

## 5. Conclusion and future perspectives

With the aging of the population, the incidence of cerebrovascular diseases has increased sharply, which has caused a heavy health and economic burden to society. Cerebrovascular diseases involve different cell types, etiologies, signaling processes, which makes research on them intricate. Continued efforts are required to better understand the pathophysiological mechanisms of cerebrovascular diseases and to investigate effective therapeutic target. In both clinical and basic research, there is a large body of evidence linking ceramide to the progression and severity of cerebrovascular diseases, including stroke and CVSD. To the best of our knowledge, there are few studies of ceramides in other types of cerebrovascular diseases, such as intracranial aneurysms. However, some studies have investigated the relationship between ceramides and non-cerebral vascular diseases, such as abdominal aortic aneurysms (Folkesson et al., 2017; Okrzeja et al., 2022). In the future, ceramides may become potential therapeutic targets for a variety of vascular diseases, including but not limited to cerebrovascular diseases. In general, the accumulation of endogenous long-chain ceramide is accompanied by the progression of cerebrovascular diseases, and interference with ceramide synthesis by either inhibiting the *de novo* synthesis pathway or sphingomyelinase activity might be beneficial for brain injury. Due to a variety of cell types involved in the pathogenesis of cerebrovascular diseases, ceramide might affect the responses of the different types of cells through different downstream signaling pathways. Therefore, in-depth studies are required to elucidate the specific role of ceramide in specific cell types in the damage and subsequent neurorepair processes associated with cerebrovascular diseases. This will allow the discovery of novel and groundbreaking therapeutic strategies to ameliorate cerebrovascular diseases that may result from accumulation in ceramide.

## Author contributions

ZZ and CL conceived and designed the study. HY and BZ wrote the manuscript. All authors listed have made a substantial, direct, and intellectual contribution to the work, and approved it for publication.
